# Evolutionary Analysis of Structural Protein Gene VP1 of Foot-and-Mouth Disease Virus Serotype Asia 1

**DOI:** 10.1155/2015/734253

**Published:** 2015-02-22

**Authors:** Qingxun Zhang, Xinsheng Liu, Yuzhen Fang, Li Pan, Jianliang Lv, Zhongwang Zhang, Peng Zhou, Yaozhong Ding, Haotai Chen, Junjun Shao, Furong Zhao, Tong Lin, Huiyun Chang, Jie Zhang, Yonglu Wang, Yongguang Zhang

**Affiliations:** State Key Laboratory of Veterinary Etiological Biology, National Foot and Mouth Disease Reference Laboratory, Key Laboratory of Animal Virology of Ministry of Agriculture, Lanzhou Veterinary Research Institute, Chinese Academy of Agricultural Sciences, Lanzhou 730046, China

## Abstract

Foot-and-mouth disease virus (FMDV) serotype Asia 1 was mostly endemic in Asia and then was responsible for economically important viral disease of cloven-hoofed animals, but the study on its selection and evolutionary process is comparatively rare. In this study, we characterized 377 isolates from Asia collected up until 2012, including four vaccine strains. Maximum likelihood analysis suggested that the strains circulating in Asia were classified into 8 different groups (groups I–VIII) or were unclassified (viruses collected before 2000). On the basis of divergence time analyses, we infer that the TMRCA of Asia 1 virus existed approximately 86.29 years ago. The result suggested that the virus had a high mutation rate (5.745 × 10^−3^ substitutions/site/year) in comparison to the other serotypes of FMDV VP1 gene. Furthermore, the structural protein VP1 was under lower selection pressure and the positive selection occurred at many sites, and four codons (positions 141, 146, 151, and 169) were located in known critical antigenic residues. The remaining sites were not located in known functional regions and were moderately conserved, and the reason for supporting all sites under positive selection remains to be elucidated because the power of these analyses was largely unknown.

## 1. Introduction

Foot-and-mouth disease (FMD) is highly contagious, typically infecting cloven-hoofed animals which can survive in animal products for a long time with severe economic consequences [1]. FMD is caused by a single-stranded positive-sense RNA virus that belongs to the genus* Aphthovirus* of the family Picornaviridae. As a typical RNA virus, FMDV virus shows high mutation frequencies reflected in rates of RNA genome evolution [2]. The FMDV RNA genome has a single large open reading frame encoding a polyprotein which can generate many different precursors during the processing of the polyprotein. After a series of cleavages, ultimately the precursors produce more than 14 mature proteins (four structural proteins VP1, VP2, VP3, and VP4 and ten nonstructural proteins L, 2A, 2B, 2C, 3A, 3B1, 3B2, 3B3, 3C, and 3D) and partial cleavage intermediates. Among the structural proteins, VP1 is exposed on the capsid surface [3, 4] and plays an essential role in forming the virus particles. The VP1 protein is highly polymorphic and carries the virus major neutralizing antigenic sites [5, 6]. Over the years, the partial or complete VP1 coding sequence has been used in the molecular epidemiological investigation, the development of engineering vaccine, and the establishment of diagnostic methods and to trace the origin and the spread of FMDV also for typing and subtyping of the virus [7–11]. So the partial and full VP1 nucleotide sequences are the preferred region for comparison of FMDV isolates strains.

The high genetic and phenotypic variability of FMDV, which occurs as multiple noncrossprotective virus serotypes, is divided into seven immunologically different serotypes (A, O, C, Asia 1, and SAT1-3) and multiple topotypes. With the widespread application of phylogenetic techniques, genetic classifications become more suitable for serological classification and subtype classification of FMDV. In addition, these phylogenetic techniques permit us to trace the origin of FMD outbreaks and have offered the possibility of more detailed analyses of outbreak strains [12]. Of these serotypes, type Asia 1, first detected in samples collected in India in 1951, is mostly endemic in Asia [13], although serotype Asia 1 caused an outbreak in Greece in 1984 and 2000 on the border with Turkey [14, 15]. The primary serotype-endemic region for FMDV serotype Asia 1 before 2003 is mainly in Southern Asia (India, Afghanistan, Iran, Pakistan, Bhutan, Turkmenistan, and Bangladesh), where outbreaks occur regularly [13, 16]. However, after 2003, serotype Asia 1 viruses spread farther north; outbreaks were first reported to occur in India, Pakistan, Iran, Afghanistan, and then in Kyrgyzstan and Tajikistan [17–19]. Subsequently in 2005, Asia had experienced widespread outbreaks of FMD due to serotype Asia 1. The appearance of this serotype in several provinces of China, Hong Kong, Mongolia, Russia, Myanmar, and Vietnam suggested that Asia 1 had spread across a wide geographic area in Asia [14, 20, 21]. Later in 2006, the incidence of Asia 1 had declined, although records of new outbreaks had been reported in China and Vietnam. However, serotype Asia 1 reported in South Asia accounts for significant proportion of the outbreaks during 2007–2012, especially in Pakistan, Afghanistan, and India [22–24].

Many studies [25–27] have been conducted which evaluated the selection and evolutionary analysis of FMDV type Asia 1 virus isolated before 2002. The aim of this study was to undertake a comprehensive and global phylogenomic analysis of 377 VP1 coding sequences collected up until 2012 and estimate selection pressure, substitution rates, and divergence times of the virus.

## 2. Materials and Methods

### 2.1. VP1 Sequence Datasets

All of these complete reference VP1 encoding sequences (*n* = 377) were obtained from National Center for Biotechnology Information (NCBI) and the FAO World Reference Laboratory for Foot and Mouth Disease (WRLFMD) which have previously been published or unpublished (Supplementary Table in Supplementary Material available online at http://dx.doi.org/10.1155/2015/734253). Four vaccine strains, IND 63/72, IND 491/97, Shamir/89, and Tajikistan/USSR/64, were used in this study. Further phylogenetic analyses were utilized for VP1 coding sequence alone. The VP1 sequences of FMDV were aligned using the Muscle algorithm available in MEGA 5.0 with the default parameters [28]. jModelTest 2.0.2 [29] was used to perform statistical selection of best-fit models of a total of 88 distinct models for nucleotide substitution. The lowest Akaike information criterion (AIC) value was considered the most appropriate model of nucleotide substitution for tree building analyses. The optimal model selected by jModelTest was GTR+I+G. Then, on the basis of the best-fit model predicted by jModelTest 2.0.2, an unrooted maximum likelihood phylogenetic tree included four rate categories was constructed using the MEGA 5.0, and the robustness of the tree topology was assessed with 1000 bootstrap replicates and all parameters were estimated from the data. Gamma distributed with invariant sites (G+I) was used to model evolutionary rate differences among sites. Finally, the phylogenetic trees were displayed using FigTree. In addition, the neighbor-joining phylogenetic relationship among 377 serotype Asia 1 viruses was also reconstructed by MEGA 5.0 under Kimura 2-parameter nucleotide substitution model.

### 2.2. Selection Analysis

Overall and site specific selection pressures were measured using a maximum likelihood method available in the HyPhy package through Datamonkey server (http://www.datamonkey.org/). The ratio of nonsynonymous (dN) to synonymous (dS) substitutions per site (dN/dS) was compared to obtain a more accurate estimation of selection pressures. Sites where dN/dS > 1 were received as positively selected, whereas sites where dN/dS < 1 indicated negative selection. Of the 377 FMDV VP1 encoding sequences, the program removed 68 sequences with 100% sequence identity, leaving 309 unique genes for selection analysis. The nucleotide substitution model, 012230, was calculated using the internal model selection function and the Akaike information criterion (AIC) [30]. Sites of positive selection were conducted using single likelihood ancestral counting (SLAC), fixed effect likelihood (FEL), internal branch FEL (IFEL), and mixed effects model of evolution (MEME).

### 2.3. Determination of Nucleotide Substitution Rates

Rates of phylogeny and molecular evolution were simultaneously estimated using the Bayesian Evolutionary Analysis by Sampling Trees program version 1.7.5 (BEAST), a commonly used Bayesian Markov chain Monte Carlo (MCMC) implementation. Removed four taxa (lineage A, B, C, and D) including the vaccine related strains; the remaining 319 sequences were calculated during the analysis. The GTR model of nucleotide substitution with gamma-distributed rates among sites and a proportion of invariant sites was applied. An exponential population growth model and a relaxed exponential uncorrelated molecular clock branch rate model were used to examine temporal origin of each clade. For our dataset, we initially set the MCMC chain length to 100 million (burn-in 10%) with parameters sampled every 2000 steps. All test parameters were examined using Tracer v1.5, including estimated sample sizes (ESS) and 95% highest posterior density (HPD) intervals. The program TreeAnnotator 1.75 was applied for obtaining an estimate of the phylogenetic tree.

## 3. Results

### 3.1. Phylogenetic Analysis of VP1 Genes

**Figure 1:**
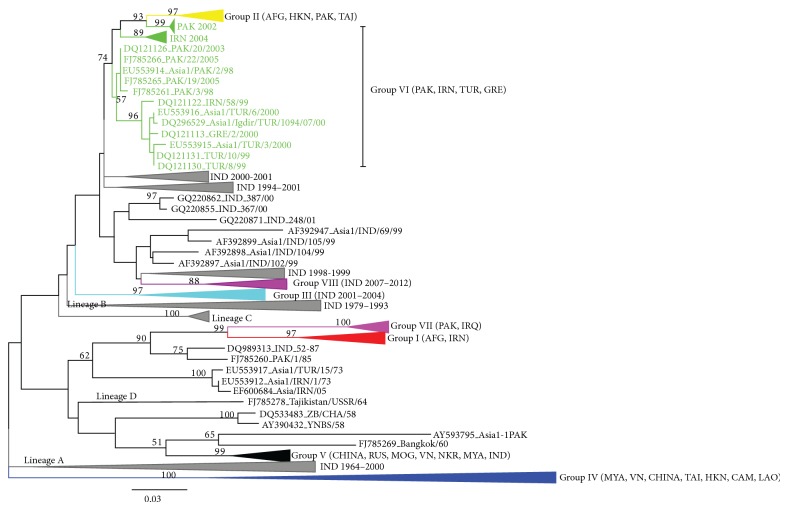
Midpoint-rooted maximum likelihood phylogenetic tree for the nucleotide sequences of the complete VP1 gene of serotype Asia 1 FMDV. Genotypes, designated as I–VIII, are shown in different colors. Numbers at nodes indicate the bootstrap values based on the maximum likelihood analysis of 1000 replicates. Scale bar (0.05) represents substitutions per nucleotide position.

Two phylogenetic algorithms were used to construct phylogenetic trees. The maximum likelihood (ML) tree is shown in Figure 1. All the 377 isolates from Asia are further subdivided into eight groups (groups I–VIII). Groups I, IV, V, and VII appeared to be responsible for most of the outbreaks which occurred in South, West, and Central Asia, whereas groups II, III, VI, and VIII were represented by the virus collected in East, North, and Southeast Asia. Strains, collected in Iran (2001 and 2004) and Afghanistan (2001), formed group I which was closely related to virus reported in India (IND_52-87) and Pakistan (PAK/1/85). Viruses of groups II (collected from Afghanistan, Pakistan, Tajikistan, Kyrgyzstan, and Hong Kong) and VI (collected from Pakistan, Iran, Greece, and Turkey) were defined and recognized by Valarcher et al. [16]. However, 13 strains collected in Afghanistan in 2009 were clustered in group II and had 96–98% nucleotide identity with remaining viruses of group II. Viruses of group III were mainly collected in India, which might suggest that this group was endemic in India. Viruses in group IV were mainly found in Southeast Asia during 2005 and 2006 that were related to virus originating from Hong Kong in the 1970s. The isolates, collected in China, Russia, Mongolia, Democratic People's Republic of Korea, Myanmar, and Vietnam in 2005–2007, belonging to group V, were clustered closely with virus from India collected in 1976, 1980, and 1981 in accordance with earlier publications. One new strain in a novel group VII, collected in Iraq (named Slemani/IRQ/2012), had 95%–96% nucleotide identity with virus collected from Pakistan during 2008-2009. Group VII was supported by bootstrap values of 100%. Recently, 43 isolates collected in India during 2007–2012 were designated as group VIII, though this excluded virus of 2005 and 2006. However, viruses isolated before 2000 showed extensive antigenic variation and were unclassified. Though these groups of viruses had been in circulation in India for some time before 2000, a similar epidemic had not broken out for a good many years.

The ML tree concluded in our analysis simultaneously revealed four monophyletic clusters with significant samples termed here as lineages A, B, C, and D. 29 isolates collected during 1964–2000 in India formed lineage A, including the currently used vaccine strain, IND63/1972. Indian isolates reported in 1979–1993 including a vaccine virus (IND 491/97, isolated during 1985) and one isolate (IND 4/2004) formed a monophyletic lineage, which was named lineage B. Vaccine strains each from Israel (Shamir/89) and Tajikistan (Tajikistan/USSR/64) formed lineages C and D, respectively.

### 3.2. Selection Analysis

The nature of selection on VP1 protein genes of serotype Asia 1 FMDV was characterized by calculating the ratio of nonsynonymous (dN) to synonymous (dS) substitutions using ML approach. Analysis of 211 amino acid sequences for positively selected sites showed the average *ω* value was 0.17 using the SLAC method. At a significance level of 0.1, SLAC reported one positively selected site (codon 47), increased significance level to 0.25 and retabulated the results to find that four codons (47, 169, 170, and 173) had *P* values for positive selection in the 0.1–0.25 range. SLAC tended to be a very conservative test and hence the actual rate of false positives could be much lower than the significance level [31]. We also used FEL, IFEL, and MEME methods to measure site specific selection pressures. The FEL and IFEL methods simply identified 1 positively selected site (position 47); MEME model which detects both diversifying and, importantly, episodic selection at individual sites identified 20 residues as under positive selection pressure with a significance level of 0.05 (Table 1). The vast majority of residues were under strong purifying selection.

### 3.3. Rates of Molecular Evolution

**Figure 2:**
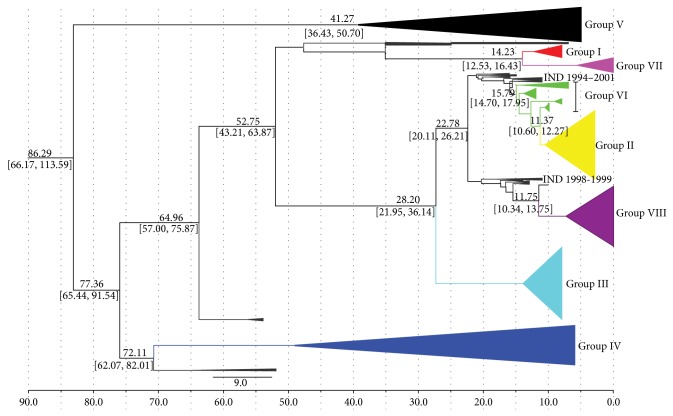
Chronogram and maximum clade credibility (MCC) tree of 3199 complete VP1 gene sequences of FMDV serotype Asia 1 generated using BEAST. The major eight groups (I–VIII) of viruses have circulated in Asia. The analysis demonstrates that mean divergence time estimates with 95% highest posterior density (HPD) credible intervals are shown in italics.

Rates of nucleotide substitution for VP1 gene sequences were using Bayesian MCMC approach implemented in the BEAST program. To calculate the rate of evolution in Asia 1 virus, isolates spanning a period of 59 years (1954 to 2012) were selected. Though lineages A, B, C, and D formed individual monophyletic clusters, they were excluded in the calculation as including the vaccine strains and some strains epidemiologically related to the vaccine virus which might lead to erroneous results. The mean rate of mutation in VP1 region was estimated at 5.745 × 10^−3^ nt substitutions per site, per year (95% HPD 4.923 × 10^−3^–6.639 × 10^−3^). On the basis of divergence time analyses, we infer that the TMRCA of 319 Asia 1 virus is 86.29 (95% HPD 66.17–113.59) (Figure 2). The estimated mean divergence time of groups IV and V was 72.11 (95% HPD 62.07–82.01) and 41.27 (95% HPD 36.43–50.74), respectively. Our analyses demonstrate that a FMDV gave rise to two primary diversification events that diverged approximately 52.75 years ago; the first diversification event generated groups II, III, VI, and VIII, whereas the second gave rise to groups I and VII (14.23 years ago). The common ancestor of groups III, VI, II, and VIII existed around 28.20 (95% HPD 21.95 and 36.14), 15.79 (95% HPD 14.70 and 17.95), 11.37 (95% HPD 10.60 and 12.27), and 11.75 (95% HPD 10.34 and 13.75) years ago, respectively.

## 4. Discussion

The recent years, 13 countries in Asia were reported to have maximum numbers of outbreaks due to serotype Asia 1. Highest numbers of outbreaks were recorded during 2000–2004 and the outbreaks of this serotype had increased from Southern Asia (Afghanistan, India, Iran, and Pakistan) to their neighbouring states (Kyrgyzstan and Tajikistan). Interestingly, although they are geographically close to India, the contemporary isolates belong to different groups. Various factors like selection pressure or stochastic events might explain the association. As described previously, FMDV serotype Asia 1 was classified into 6 different groups (I–VI) [26]. Further, viruses collected from Pakistan during 2008–2009 were designated as a novel group (VII), which show about 91% nucleotide identity with group I. Of all these groups, nucleotide identity within VP1 nucleotide sequences was 95%–100%. Afterwards, there was another mainly upsurge of this serotype virus in the years 2007–2012 in India, a novel genetic group designated as group VIII [32]. The appearances of the Asia 1 serotype viruses belonging to group V which were responsible for these outbreaks collected in the Eastern, Southern, and Western regions of China in 2005 and 2006, Mongolia in 2005, Russia in 2005, and Vietnam in 2007 were closely related to each other (less than 1% difference) and to viruses from India isolated in 1980 and 1981 (less than 2% difference). Isolate of Democratic People's Republic of Korea collected in 2007 shared highest nucleotide identity (98.3%) to Russian isolates. This finding suggests that a single group of Asia 1 viruses could be spreading throughout many countries. Thirteen factors in the emergence of infectious diseases were elucidated and the four broad domains were genetic and biological factors, physical environmental factors, ecological factors, and social, political, and economic factors [33]. It is speculated that economic factors may play a major role in the virus circulating among these countries.

Through observation in phylogenetic studies of DENV, it was found that lineage turnover followed two forms [34]. The first form is sequential clade replacement, eliminating deleterious mutations by purifying selection, which result in phylogenetic trees with a strong temporal topology [35]. Another form presents dramatic clade replacement which showed that an entire clade of virus that persisted in a particular region for a number of years dropped dramatically in frequency and was replaced by a new clade of virus. We observed that the virus belonging to group II collected in Afghanistan in 2003 and 2009 had a ladder-like structure, although it was less pronounced than the hemagglutinin gene of human influenza A virus [36]. In particular, our estimated of overall selection pressures demonstrated that the purifying selection was shaping serotype Asia 1 FMDV evolution. It has become clear that the Afghanistan strains follow sequential clade replacement. Most notably, through the analysis of sampled data, we found a dramatic mode of lineage turnover on two occasions in India and Pakistan. The India strains (collected in 2000–2004) and Pakistan strains (collected in 2002–2005) persisted in particular countries for a period of time and then were replaced by groups VIII and VII, respectively. Although the evolutionary processes for these events remain uncertain, dramatic population bottlenecks or selective pressures might have played a role.

Evidence provided support for the presence of positively selected sites in the capsid-coding genes, which were specifically identified at known critical antigenic residues [37, 38]. Earlier studies had identified 13 mutable neutralization antigenic sites (48, 59, 140, 141, 146, 151, 153, 169, 182, 192, 202, 207, and 210) to be antigenically significant in FMDV serotype Asia 1. The sites based on our analysis were located in B-C loop, G-H loop, and the C-terminus of VP1, respectively. Simultaneously most of antigenic sites are located in G-H loop. These regions were crucial to the antigenicity of FMDV. The major neutralizing antigenic site was located in G-H loop (encompassing residues 140–160) [39]. It was also found that epitope _194_(TTQDRRKQEIIAPEKQTL)_211_ located in the terminal regions of C showed a high variability index, as determined by Zhang et al. [40]. Of the positive selection positions identified, positions 141, 146, 151, and 169 were located in previously characterised functionally independent neutralizing antigenic sites. Analysis of the four sites under positive selection indicates that this selective advantage leads to the major antigenic variants [141 (S/P/T/R/E/A), 146 (M/L/A/T), 151 (Q/R/G/K), and 169 (E/D/T/N)]. Significant mutation proneness was also found for position 96 [41], where more than four amino acid replacements were observed [96 (D/T/A/V)]. The mutations at positions 140 (T/P/A/S), 141(S/P/T), and 142 (R/W/Q/M/L) that under positive selection pressure were noticed in Indian field isolates were covered during 1964–2012 [32]. However, position 142 is moderately conserved in our work, and 2 positions (140, 141) are also frequently substituted. Another 5 positions (47, 138, 139, 154, and 170) were frequently substituted and more than five amino acid replacements were observed. The remaining sites were not located in known functional regions and were moderately conserved, and the reason to support all sites under positive selection remains to be elucidated because the power of these analyses was largely unknown. It was found that the positive selection by interaction with the immune mechanism or infection process may play a major role in FMDV evolution [32]. Previous research had suggested that the antigenic shift and wide host range appeared to influence the virus evolution [41]. The major neutralizing antigenic sites are extremely important due to reason that they are involved in binding to target cell receptors and helping virus entry into susceptible cells. We believe that immune response may be the dominant driving force in the adaptive evolution in type Asia 1 virus.

Evolutionary rates for RNA viruses range between 10^−1^ and 10^−4^ substitutions per nucleotide per year. The rate of evolutionary changes for serotype Asia 1 virus collected up until 2012 was consistent with the characteristic of RNA virus. The values calculated in early analyses indicated that the mean evolutionary rate was 2.48 × 10^−3^ substitutions per site per year of all serotypes of FMDV [27]. For the seven serotypes, the evolutionary rate of Asia 1 (6.32 × 10^−3^) is faster than the others [27]. Further, the rate calculated in this study is in accordance with an recently analyses demonstrated that the overall evolutionary rate of Indian isolates was 5.871 × 10^−3^ substitutions per site per year [32]. However, the value is different from that observed (synonymous and nonsynonymous substitution rate estimated to be 2.7 × 10^−2^ and 1.1 × 10^−3^) in the case of the Asia 1 virus isolated in India between 1985 and 1999 [26]. But we must emphasize the fact that the evolution of the foot-and-mouth disease virus depends on the time period and the genomic segment [42]. And these analyses included limited number of Asia 1 isolates covering a limited period of years which might lead to inaccurate results.

From the Bayesian coalescent analysis, we carry on research on FMDV Asia 1 circulating in Asia. One study shows that the ancestors of Asia 1 isolates examined between 1954 and 1999 existed at approximately 96 years ago (95% HPD 54–161) [27]. Another time scale analysis indicated that the most recent common ancestor of lineages C and D and genotype II is believed to exist during 1974.7 and 1984.1, 1988.4 and 1992.2, and 1962 and 1978, respectively [32]. Our estimates indicate that a similar age of common ancestor for Asia 1 isolates could be 86.29 (95% HPD 66.17–113.59). The common ancestor of the six groups (I, II, III, VI, VII, and VIII) existed around 52.75 years ago with 95% confidence intervals of 43.21 to 63.87. Earliest Asia 1 sequence in our analysis was detected in Pakistan in 1954 as a result of lacking available Indian sequences during 1951–1964. Apart from the vaccine related strains, our MRCA estimated all available variants.

In summary, we have a comprehensive understanding of evolutionary process of FMDV serotype Asia 1. The VP1 gene sequences of serotype Asia 1, in comparison to the other serotypes of FMDV, exhibit a high mutation rate. It is obvious that Asia 1 strains circulating in Asia are under strong negative selection, indicating that most mutations are synonymous. We believe that the virus under negative selection provides the potential to produce escape variants in the face of widespread vaccination or other environmental factors. Analysis of FMDV serotype Asia 1 genetic evolution may aid in the development of vaccine research and avoid viral escape mutants.

## Supplementary Material

History of the FMDV serotype Asia1 field isolates used in the study and it is included all virus strains background information.

## Figures and Tables

**Table 1:** Selection pressure estimates for VP1 region of 319 FMDV serotype Asia 1 strains. Antigenically critical sites and significant mutation sites are in bold.

Model	SLAC(*P* values 0.1–0.25)	FEL/IFEL(*P* values 0.1–0.25)	MEME(*P* values 0.05)
Positively selected sites	**47**, **169**, **170**, 173	**47**	6, 26, 27, **47**, 68, **96**, 97, 101, 117, 129, 130, 137, **138**, **139**, **141**, **146** 150, **151**, **154**, 205
