# Supplementary material for: Evolutionary Analysis of Structural Protein Gene VP1 of Foot-and-Mouth Disease Virus Serotype Asia 1
Source: ScientificWorldJournal. 2015 Feb 22;2015:734253. doi: 10.1155/2015/734253 (PMC4352495; doi:10.1155/2015/734253)
Supplement: Supplementary file 1 — History of the FMDV serotype Asia1 field isolates used in the study and it is included all virus strains background information. [file 734253.f1.pdf]

Supplementary Table History of the FMDV serotype Asia1 field isolates used in the study.

	Virus Isolate	Place of Isolation	Data of collection	Species	Reference	Accession No
1	AFG/116/2004	Afghanistan	2004	Bovine	Schumann et al,. 2008	EF457993
2	AFG/138/2004	Afghanistan	2004	Bovine	Schumann et al,. 2008	EF457994
3	AFG/22/2003	Afghanistan	2003	Bovine	Schumann et al,. 2008	EF457987
4	AFG/24/2003	Afghanistan	2003	Bovine	Schumann et al,. 2008	EF457988
5	AFG/26/2003	Afghanistan	2003	Bovine	Schumann et al,. 2008	EF457989
6	AFG/33/2003	Afghanistan	2003	Bovine	Schumann et al,. 2008	EF457990
7	AFG/40/2003	Afghanistan	2003	Bovine	Schumann et al,. 2008	EF457991
8	AFG/44/2003	Afghanistan	2003	Bovine	Schumann et al,. 2008	EF457992
9	Amursky/Russia/2005	Russia	2005	NK	Valarcher et al,. 2005	DQ121401
10	BAM/AFG/L1/2009	Afghanistan	2009	Cattle	Jamal et al,. 2011	HQ439194
11	BAM/AFG/L2/2009	Afghanistan	2009	Cattle	Jamal et al,. 2011	HQ439195
12	BAM/AFG/L2823/2009	Afghanistan	2009	Cattle	Jamal et al,. 2011	HQ439203

13	BAM/AFG/L2824/2009	Afghanistan	2009	Cattle	Jamal et al,. 2011	HQ439204
14	BAM/AFG/L2825/2009	Afghanistan	2009	Cattle	Jamal et al,. 2011	HQ439205
15	BAM/AFG/L3/2009	Afghanistan	2009	Cattle	Jamal et al,. 2011	HQ439196
16	BAM/AFG/L-590/2009	Afghanistan	2009	NK	Belsham et al,. 2011	HQ113233
17	BAM/AFG/L591/2009	Afghanistan	2009	NK	Jamal et al,. 2011	HQ439199
18	BAM/AFG/L638/2009	Afghanistan	2009	Cattle	Jamal et al,. 2011	HQ439197
19	BAM/AFG/L639/2009	Afghanistan	2009	Cattle	Jamal et al,. 2011	HQ439198
20	BAM/AFG/L640/2009	Afghanistan	2009	Cattle	Jamal et al,. 2011	HQ439200
21	BAM/AFG/L641/2009	Afghanistan	2009	Cattle	Jamal et al,. 2011	HQ439201
22	BeiJing/CHA/2005	China	2005	Bovine	Valarcher et al,. 2009	EF185303
23	BR/Myanmar/1/06	China	2006	Bovine	Zhang et al., unpub. data	EU091342
24	BR/Myanmar/2/06	China	2006	Bovine	Zhang et al., unpub. data	EU091343
25	BR/Myanmar/3/06	China	2006	Bovine	Zhang et al., unpub. data	EU091344
26	BR/Myanmar/4/06	China	2006	Bovine	Zhang et al., unpub. data	EU091345
27	BR/Myanmar/5/06	China	2006	Bovine	Zhang et al., unpub. data	EU091346

28	BR/Myanmar/6/06	China	2006	Bovine	Zhang et al., unpub. data	EU091347
29	BR/Myanmar/7/06	China	2006	Bovine	Zhang et al., unpub. data	EU091348
30	GanSu/CHA/2005	China	2005	Bovine	Valarcher et al,. 2009	EF185304
31	HeB/CHA/1/2005	China	2005	Bovine	Valarcher et al,. 2009	EF187274
32	HeB/CHA/2/2005	China	2005	Bovine	Valarcher et al,. 2009	EF187273
33	HKN/1/2005	Hongkong	2005	Cattle	Valarcher et al,. 2005	DQ121114
34	HKN/2/2005	Hongkong	2005	Cattle	Valarcher et al,. 2005	DQ121115
35	HKN/3/2005	Hongkong	2005	Cattle	Valarcher et al,. 2009	FJ785235
36	HKN/4/2005	Hongkong	2005	Cattle	Valarcher et al,. 2009	FJ785236
37	HKN/5/2005	Hongkong	2005	Cattle	Valarcher et al,. 2009	FJ785237
38	HKN/6/2005	Hongkong	2005	Cattle	Valarcher et al,. 2009	FJ785238
39	HKN/7/2005	Hongkong	2005	Cattle	Valarcher et al,. 2009	FJ785239
40	HKN/8/2005	Hongkong	2005	Cattle	Valarcher et al,. 2009	FJ785240
41	IND/12/2007	India	2007	Bovine	Sanyal et al,.2010	HQ224553
42	IND/121/2007	India	2007	Bovine	Sanyal et al,.2010	HQ224554
43	IND/137/2008	India	2008	Bovine	Sanyal et al,.2010	HQ224561
44	IND/227/2007	India	2007	Bovine	Sanyal et al,.2010	HQ224555
45	IND/32/2008	India	2007	Bovine	Sanyal et al,.2010	HQ224556
46	IND/93/2008	India	2007	Bovine	Sanyal et al,.2010	HQ224557
47	IND/95/2008	India	2007	Bovine	Sanyal et al,.2010	HQ224558
48	IND/96/2008	India	2007	Bovine	Sanyal et al,.2010	HQ224559

48	IND/97/2008	India	2007	Bovine	Sanyal et al,.2010	HQ224560
50	IND/147/2004	India	2003	Bovine	Valarcher et al,. 2009	FJ785294
51	IND/150/2004	India	2003	Bovine	Valarcher et al,. 2009	FJ785295
52	IND/153/2004	India	2003	Bovine	Valarcher et al,. 2009	FJ785296
53	IND/156/2004	India	2003	Bovine	Valarcher et al,. 2009	FJ785297
54	IND/158/2004	India	2003	Bovine	Valarcher et al,. 2009	FJ785298
55	IND/168/2004	India	2004	Bovine	Valarcher et al,. 2009	FJ785299
56	IND/322/2004	India	2004	Bovine	Valarcher et al,. 2009	FJ785300
57	IND/325/2004	India	2004	Bovine	Valarcher et al,. 2009	FJ785301
58	IND/327/2004	India	2004	Bovine	Valarcher et al,. 2009	FJ785302
58	IND/328/2004	India	2004	Bovine	Valarcher et al,. 2009	FJ785303
60	IND/389/2004	India	2004	Bovine	Valarcher et al,. 2009	FJ785304
61	IND/4/2004	India	2003	NK	Valarcher et al,. 2009	FJ785293
62	IND/763/2003	India	2003	Bovine	Valarcher et al,. 2005	FJ785292
63	IND/114/2012	India	2012	Cattle	Subramaniam et al,.2013	KF570668
64	IND/114/2004	India	2004	Bovine	Valarcher et al,. 2005	DQ101239
65	IND/115/2012	India	2012	Buffalo	Subramaniam et al,.2013	KF570669
66	IND/118/2012	India	2012	Cattle	Subramaniam et al,.2013	KF570670
67	IND/119/2012	India	2012	Cattle	Subramaniam et al,.2013	KF570671
68	IND/120/2012	India	2012	Buffalo	Subramaniam et al,.2013	KF570672
69	IND/121/2012	India	2012	Cattle	Subramaniam et al,.2013	KF570673
70	IND/125/2011	India	2011	Cattle	Subramaniam et al,.2013	KF570661
71	IND/127/2011	India	2011	Cattle	Subramaniam et al,.2013	KF570662
72	IND/129/2011	India	2011	Cattle	Subramaniam et al,.2013	KF570663

73	IND/133/2011	India	2011	Cattle	Subramaniam et al,.2013	KF570664
74	IND/135/2012	India	2012	Cattle	Subramaniam et al,.2013	KF570674
75	IND/148/2011	India	2011	Cattle	Subramaniam et al,.2013	KF570665
76	IND/16/2012	India	2011	Cattle	Subramaniam et al,.2013	KF570666
77	IND/165/2004	India	2004	Bovine	Valarcher et al,. 2005	DQ101238
78	IND/175/2004	India	2004	Bovine	Valarcher et al,. 2005	DQ101237
79	IND/205/2010	India	2010	Cattle	Subramaniam et al,.2013	KF570658
80	IND/248/2009	India	2009	Cattle	Subramaniam et al,.2013	KF570654
81	IND/268/2004	India	2004	Bovine	Valarcher et al,. 2005	DQ101236
82	IND/283/2012	India	2012	Cattle	Subramaniam et al,.2013	KF570675
83	IND/285/2012	India	2012	Cattle	Subramaniam et al,.2013	KF570676
84	IND/288/2012	India	2012	Cattle	Subramaniam et al,.2013	KF570677
85	IND/291/2012	India	2012	Cattle	Subramaniam et al,.2013	KF570678
86	IND/292/2012	India	2011	Cattle	Subramaniam et al,.2013	KF570679
87	IND/294/2012	India	2012	Cattle	Subramaniam et al,.2013	KF570680
88	IND/300/2012	India	2012	Cattle	Subramaniam et al,.2013	KF570681
89	IND/303/2012	India	2012	Cattle	Subramaniam et al,.2013	KF570682
90	IND/305/2012	India	2012	Cattle	Subramaniam et al,.2013	KF570683
91	IND/306/2012	India	2012	Cattle	Subramaniam et al,.2013	KF570684
92	IND/327/2009	India	2009	Cattle	Subramaniam et al,.2013	KF570655
93	IND/341/2008	India	2008	Cattle	Subramaniam et al,.2013	KF570653
94	IND/388/2004	India	2004	Bovine	Valarcher et al,. 2005	DQ101235
95	IND/65/2010	India	2010	Cattle	Subramaniam et al,.2013	KF570657
96	IND/673/2003	India	2003	Bovine	Valarcher et al,. 2009	DQ101241

97	IND/7/2011	India	2010	Cattle	Subramaniam et al,.2013	KF570659
98	IND/762/2003	India	2003	Bovine	Valarcher et al,. 2009	DQ101240
99	IND/78/2011	India	2011	Cattle	Subramaniam et al,.2013	KF570660
100	IND/787/2009	India	2009	Cattle	Subramaniam et al,.2013	KF570656
101	IRN/10/2004	Iran	2004	Bovine	Valarcher et al,. 2005	DQ121119
102	IRN/25/2004	Iran	2004	Bovine	Valarcher et al,. 2005	DQ121120
103	IRN/30/2004	Iran	2004	NK	Valarcher et al,. 2009	FJ785246
104	IRN/31/2004	Iran	2004	NK	Valarcher et al,. 2005	DQ121121
105	JiangSu/CHA/1/2005	China	2005	Bovine	Valarcher et al,. 2009	EF149009
106	JiangSu/CHA/2/2005	China	2005	Bovine	Valarcher et al,. 2005	DQ156527
107	KAB/AFG/L642/2009	Afghanistan	2009	Cattle	Jamal et al,. 2011	HQ439202
108	Khabarovsk/RUS/2005	Russia	2005	Bovine	Valarcher et al,. 2009	FJ785267
109	KRG/1/2004*	Kyrgyzstan	2004	NK	Valarcher et al,. 2009	FJ785248
110	KRG/1/2004	Kyrgyzstan	2004	NK	Valarcher et al,. 2009	FJ785247
111	KRG/2/2004	Kyrgyzstan	2004	NK	Valarcher et al,. 2009	FJ785249
112	MOG/05	Mongolia	2005	Cattle	Lee et al,. 2009	EF614458
113	MOG/2005	Mongolia	2005	Bovine	Valarcher et al,. 2009	FJ785252
114	MYA/1/05	Myanmar	2005	NK	Valarcher et al,. 2009	FJ785257
115	MYA/1/2005	Myanmar	2005	NK	Valarcher et al,. 2009	FJ785258
116	NKR/2007	North Korea	2007	Bovine	Valarcher et al,. 2009	FJ785259
117	PAK/1/2004	Pakistan	2004	Buffalo	Valarcher et al., 2005	DQ121128

118	PAK/19/2005	Pakistan	2005	Bovine	Valarcher et al., 2009	FJ785265
119	PAK/2/2004	Pakistan	2004	Cattle	Valarcher et al., 2005	FJ785264
120	PAK/20/2003	Pakistan	2003	NK	Valarcher et al., 2005	DQ121126
121	PAK/22/2005	Pakistan	2005	Cattle	Valarcher et al., 2009	FJ785266
122	PAK/69/2003	Pakistan	2003	Cattle	Valarcher et al., 2005	DQ121127
123	PD 155/2010	India	2010	Cattle	Kumar et al., 2011	JN247565
124	PD 18/2011	India	2011	Cattle	Kumar et al., 2011	JN247567
125	PD 509/2010	India	2010	Cattle	Kumar et al., 2011	JN247566
126	Prymorsky/RUS /2005	Russia	2005	Bovine	Valarcher et al,. 2009	FJ785268
127	QH/CHA/2005	China	2005	Bovine	Valarcher et al,. 2009	EF187272
128	S/IRQ/2012	Iraq	2012			JX455113
129	SIN/PAK/L2810 /2009	Pakistan	2009	Buffalo	Jamal et al,. 2011	HQ439189
130	SIN/PAK/L2811 /2009	Pakistan	2009	Buffalo	Jamal et al,. 2011	HQ439190
131	SIN/PAK/L2812 /2009	Pakistan	2009	Buffalo	Jamal et al,. 2011	HQ439191
132	SIN/PAK/L2952 /2009	Pakistan	2009	Buffalo	Jamal et al,. 2011	HQ439192
133	SIN/PAK/L2954 /2009	Pakistan	2009	Buffalo	Jamal et al,. 2011	HQ439193
134	SIN/PAK/L5/20 08	Pakistan	2008	Buffalo	Jamal et al,. 2011	HQ439187
135	SIN/PAK/L8/20 08	Pakistan	2008	Buffalo	Jamal et al,. 2011	HQ439188
136	TAJ/1/2003	Tajikistan	2003	Bovine	Valarcher et al,. 2009	FJ785270

137	Taj/1/2004	Tajikistan	2004	NK	Valarcher et al,. 2005	DQ121402
138	TAJ/2/2003	Tajikistan	2003	Bovine	Valarcher et al,. 2009	FJ785271
139	Taj/2/2004	Tajikistan	2004	NK	Valarcher et al,. 2005	DQ121403
140	TAJ/3/2003	Tajikistan	2003	Bovine	Valarcher et al,. 2009	FJ785272
141	TAJ/3/2004	Tajikistan	2004	NK	Valarcher et al,. 2009	FJ785273
142	TAJ/4/2004	Tajikistan	2004	NK	Valarcher et al,. 2009	FJ785274
143	TAJ/5/2004	Tajikistan	2004	NK	Valarcher et al,. 2009	FJ785275
144	TAJ/6/2004	Tajikistan	2004	NK	Valarcher et al,. 2009	FJ785276
145	VIT 1/06	Vietnam	2005	Bovine	Valarcher et al,. 2009	FJ785284
146	VIT 2/06	Vietnam	2005	Bovine	Valarcher et al,. 2009	FJ785286
147	VIT 3/06	Vietnam	2005	Buffalo	Valarcher et al,. 2009	FJ785288
148	VIT 4/06	Vietnam	2005	Buffalo	Valarcher et al,. 2009	FJ785290
149	VIT/10/2006	Vietnam	2005	Buffalo	Valarcher et al,. 2009	FJ785287
150	VIT/11/2006	Vietnam	2005	Buffalo	Valarcher et al,. 2009	FJ785289
151	VIT/15/2005	Vietnam	2005	Bovine	Valarcher et al,. 2009	FJ785281
152	VIT/16/2005	Vietnam	2005	Bovine	Valarcher et al,. 2009	FJ785282
153	VIT/8/2006	Vietnam	2005	Bovine	Valarcher et al,. 2009	FJ785283
154	VIT/9/2006	Vietnam	2005	Bovine	Valarcher et al,. 2009	FJ785285
155	VN/LC04/2005	Vietnam	2005	Buffalo	Le et al,.2010	GU125646
156	VN/QT/2007	Vietnam	2007	Cattle	Lee et al,. 2011	GQ452295
157	VN/QT03/2007	Vietnam	2007	Cattle	Le et al,.2010	GU125645
158	VN/QT466/2007	Vietnam	2007	Pig	Le et al,. 2011	GU582123
159	VN/QT523/2007	Vietnam	2007	Cattle	Le et al,. 2011	GU582124

160	WHN/CHA/06	China	2006	Pig	Yang et al., 2011	FJ906802
161	WNH 06	China	2006	Porcine	Wang et al., unpub. data	EU887277
162	IND/2/71(1964)	India	1964	Bovine	Valarcher et al,. 2009	FJ785241
163	IND/16/76	India	1976	NK	Valarcher et al,. 2009	FJ785242
164	AFG/2/2001	Afghanistan	2001	Bovine	Valarcher et al,. 2009	FJ785226
165	AFG/3/2001	Afghanistan	2001	Bovine	Valarcher et al,. 2009	FJ785227
166	CAM/9/80	Cambodia	1980	NK	Valarcher et al,. 2009	FJ785228
167	CAM/5/97	Cambodia	1997	Bovine	Valarcher et al,. 2009	FJ785229
168	HKN/19/74	Hong Kong	1974	Bovine	Valarcher et al,. 2009	FJ785230
169	HKN/2/75	Hong Kong	1974	Bovine	Valarcher et al,. 2009	FJ785231
170	HKN/24/75	Hong Kong	1975	Bovine	Valarcher et al,. 2009	FJ785232
171	HKN/18/76	Hong Kong	1976	Bovine	Valarcher et al,. 2009	FJ785233
172	HKN/22/80	Hong Kong	1980	Bovine	Valarcher et al,. 2009	FJ785234
173	IND 180/02	India	2002	Bovine	Valarcher et al,. 2009	FJ785291
174	IRN/11/2001	Iran	2001	Bovine	Valarcher et al,. 2009	FJ785243

175	IRN/25/2001	Iran	2001	NK	Valarcher et al,. 2009	FJ785244
176	IRN/63/2001	Iran	2001	Bovine	Valarcher et al,. 2009	FJ785245
177	MAY/8/97	Malaysia	1997	Bovine	Valarcher et al,. 2009	FJ785250
178	MAY/9/99	Malaysia	1999	Bovine	Valarcher et al,. 2009	FJ785251
179	MYA/2/97	Myanmar	1997	Bovine	Valarcher et al,. 2009	FJ785253
180	MYA/3/2000	Myanmar	2000	Bovine	Valarcher et al,. 2009	FJ785254
181	MYA/4/2000	Myanmar	2000	Bovine	Valarcher et al,. 2009	FJ785255
182	MYA/5/2000	Myanmar	2000	Bovine	Valarcher et al,. 2009	FJ785256
183	PAK/1/85	Pakistan	1985	Bovine	Valarcher et al,. 2009	FJ785260
184	PAK/3/98	Pakistan	1998	Bovine	Valarcher et al,. 2009	FJ785261
185	PAK/33/2002	Pakistan	2002	Buffalo	Valarcher et al,. 2009	FJ785262
186	PAK/34/2002	Pakistan	2002	Buffalo	Valarcher et al,. 2009	FJ785263
187	Bangkok/60	Thailand	1960	NK	Valarcher et al,. 2009	FJ785269
188	Tajikistan/USSR/64	USSR	1964	NK	Valarcher et al,. 2009	FJ785278
189	VIT/1/92	Vietnam	1992	Bovine	Valarcher et al,. 2009	FJ785280
190	IND_63/72	India	1972	Bovine	Saravanan et al., unpub. data	AY304994
191	IND 10/86	India	1986	Bovine	Gurumurthy et al., 2002	AF392901
192	IND 75/86	India	1986	Bovine	Gurumurthy et al., 2002	AF390702
193	IND 82/86	India	1986	Bovine	Gurumurthy et al., 2002	AF392914
194	IND 46/87	India	1987	Bovine	Gurumurthy et al., 2002	AF390692
195	IND 22/88	India	1988	Bovine	Gurumurthy et al., 2002	AF390685
196	IND 120/88	India	1988	Bovine	Gurumurthy et al., 2002	AF390675
197	IND 177/88	India	1988	Bovine	Gurumurthy et al., 2002	AF392904

198	IND 267/88	India	1988	Bovine	Gurumurthy et al., 2002	AF390686
199	IND 19/89	India	1989	Buffalo	Gurumurthy et al., 2002	AF390684
200	IND 45/89	India	1989	Buffalo	Gurumurthy et al., 2002	AF390691
201	IND 2/90	India	1990	Bovine	Gurumurthy et al., 2002	AF392912
202	IND 132/90	India	1990	Bovine	Gurumurthy et al., 2002	AF390676
203	IND 10/91	India	1991	Bovine	Gurumurthy et al., 2002	AF390674
204	IND 13/91	India	1991	Ovine	Gurumurthy et al., 2002	AF390677
205	IND 17/91	India	1991	Bovine	Gurumurthy et al., 2002	AF390682
206	IND 51/93	India	1993	Bovine	Gurumurthy et al., 2002	AF392923
207	IND 53/93	India	1993	Bovine	Gurumurthy et al., 2002	AF390695
208	IND 281/94	India	1994	Mithun	Gurumurthy et al., 2002	AF392910
209	IND 1/95	India	1995	Bovine	Gurumurthy et al., 2002	AF390683
210	IND 4/95	India	1995	Bovine	Gurumurthy et al., 2002	AF390693
211	IND 6/95	India	1995	Bovine	Gurumurthy et al., 2002	AF390697
212	IND 14/95	India	1995	Bovine	Gurumurthy et al., 2002	AF390678
213	IND 15/95	India	1995	Bovine	Gurumurthy et al., 2002	AF390680
214	IND 26/95	India	1995	Bovine	Gurumurthy et al., 2002	AF390687
215	IND 43/95	India	1995	Bovine	Gurumurthy et al., 2002	AF392916
216	IND 47/95	India	1995	Bovine	Gurumurthy et al., 2002	AF392921
217	IND 50/95	India	1995	Buffalo	Gurumurthy et al., 2002	AF390694
218	IND 57/95	India	1995	Bovine	Gurumurthy et al., 2002	AF390696
219	IND 33/96	India	1996	Bovine	Gurumurthy et al., 2002	AF390689
220	IND 43/96	India	1996	Bovine	Gurumurthy et al., 2002	AF390690
221	IND 70/96	India	1996	Bovine	Gurumurthy et al., 2002	AF390698

222	IND 72/96	India	1996	Bovine	Gurumurthy et al., 2002	AF390700
223	IND 73/96	India	1996	Bovine	Gurumurthy et al., 2002	AF390701
224	IND 80/96	India	1996	Bovine	Gurumurthy et al., 2002	AF390703
225	IND 81/96	India	1996	Bovine	Gurumurthy et al., 2002	AF390704
226	IND 82/96	India	1996	Bovine	Gurumurthy et al., 2002	AF390705
227	IND 89/96	India	1996	Buffalo	Gurumurthy et al., 2002	AF390706
228	IND 173/96	India	1996	Buffalo	Gurumurthy et al., 2002	AF390681
229	IND 256/97	India	1997	Bovine	Gurumurthy et al., 2002	AF392906
230	IND 294/97	India	1997	Bovine	Gurumurthy et al., 2002	AF392913
231	IND 386/97	India	1997	Bovine	Gurumurthy et al., 2002	AF392914
232	IND 388/97	India	1997	Bovine	Gurumurthy et al., 2002	AF392915
233	IND 491/97	India	1997	NK	Gurumurthy et al., 2002	AF392922
234	IND 270/98	India	1998	Bovine	Gurumurthy et al., 2002	AF392907
235	IND 271/98	India	1998	Bovine	Gurumurthy et al., 2002	AF392908
236	IND 451/98	India	1998	Buffalo	Gurumurthy et al., 2002	AF392917
237	IND 452/98	India	1998	Bovine	Gurumurthy et al., 2002	AF392918
238	IND 453/98	India	1998	Buffalo	Gurumurthy et al., 2002	AF392919
239	IND 454/98	India	1998	Buffalo	Gurumurthy et al., 2002	AF392920
240	IND 92/99	India	1999	Bovine	Gurumurthy et al., 2002	AF392925
241	IND 102/99	India	1999	Buffalo	Gurumurthy et al., 2002	AF392897
242	IND 104/99	India	1999	Buffalo	Gurumurthy et al., 2002	AF392898
243	IND 105/99	India	1999	Bovine	Gurumurthy et al., 2002	AF392899
244	IND 107/99	India	1999	Bovine	Gurumurthy et al., 2002	AF392900
245	IND 108/99	India	1999	Bovine	Gurumurthy et al., 2002	AF392902

246	IND 126/99	India	1999	Bovine	Gurumurthy et al., 2002	AF392903
247	IND 196/99	India	1999	Bovine	Gurumurthy et al., 2002	AF392905
248	IND 277/99	India	1999	Bovine	Gurumurthy et al., 2002	AF392909
249	IND 286/99	India	1999	Bovine	Gurumurthy et al., 2002	AF392911
250	IND/18/80	India	1980	Bovine	Valarcher et al,. 2005	DQ121116
251	AFG/1/2001	Afghanistan	2001	Bovine	Valarcher et al,. 2005	DQ121109
252	AFG/4/2001	Afghanistan	2001	Bovine	Valarcher et al,. 2005	DQ121110
253	BHU/27/2002	Bhutan	2002	NK	Valarcher et al,. 2005	DQ121111
254	BHU/34/2002	Bhutan	2002	NK	Valarcher et al,. 2005	DQ121112
255	GRE/2/2000	Greece	2000	NK	Valarcher et al,. 2005	DQ121113
256	IND/15/81	India	1981	Bovine	Valarcher et al,. 2005	DQ121117
257	IND 60/02	India	2002	Bovine	Valarcher et al,. 2005	DQ101243
258	IND 139/02	India	2002	Bovine	Valarcher et al,. 2005	DQ101242
259	IND 198/02	India	2002	Bovine	Valarcher et al,. 2005	DQ101244
260	IRN/58/99	India	1999	NK	Valarcher et al,. 2005	DQ121122
261	IRN/4/2001	India	2001	Bovine	Valarcher et al,. 2005	DQ121118
262	MYA/2/2001	Myanmar	2001	Bovine	Valarcher et al,. 2005	DQ121123
263	PAK/30/2002	Pakistan	2002	Buffalo	Valarcher et al,. 2005	DQ121124
264	PAK/31/2002	Pakistan	2002	Buffalo	Valarcher et al,. 2005	DQ121125
265	TAI/1/98	Thailand	1998	Buffalo	Valarcher et al,. 2005	DQ121129
266	TUR/8/99	Turkey	1999	Bovine	Valarcher et al,. 2005	DQ121130
267	TUR/10/99	Turkey	1999	Bovine	Valarcher et al,. 2005	DQ121131
268	As1/Shamir/89	Israel	1989	NK	Lee et al., unpub. data	JF739177
269	GRE/1/84	Greece	1984	Bovine	Valarcher et al,. 2008	EU553909

270	IND/8/79	India	1979	NK	Valarcher et al,. 2008	EU553910
271	WBN/117/85	India	1985	NK	Valarcher et al,. 2008	EU553911
272	IRN/1/73	Iran	1973	NK	Valarcher et al,. 2008	EU553912
273	ISR/1/57	Israel	1957	NK	Valarcher et al,. 2008	EU553913
274	PAK/2/98	Pakistan	1998	Bovine	Valarcher et al,. 2008	EU553914
275	TUR/15/73	Turkey	1973	Bovine	Valarcher et al,. 2008	EU553917
276	TUR/3/2000	Turkey	2000	Bovine	Valarcher et al,. 2008	EU553915
277	TUR/6/2000	Turkey	2000	Bovine	Valarcher et al,. 2008	EU553916
278	IND 68/92	India	1992	Bovine	Sanyal et al., unpub. Data	AF392946
279	IND 247/92	India	1992	Bovine	Sanyal et al., unpub. Data	AF392932
280	IND 49/93	India	1993	Buffalo	Sanyal et al.,2004	AF392945
281	IND 152/94	India	1994	Bovine	Sanyal et al.,2004	AF392930
282	IND 175/94	India	1994	Bovine	Sanyal et al.,2004	AF392931
283	IND 305/94	India	1994	Bovine	Sanyal et al.,2004	AF392936
284	IND 298/94	India	1994	Bovine	Sanyal et al.,2004	AF392935
285	IND 24/95	India	1995	Bovine	Sanyal et al.,2004	AF392933
286	IND 339/96	India	1996	Bovine	Sanyal et al.,2004	AF392939
287	IND 390/97	India	1997	Bovine	Sanyal et al.,2004	AF392940
288	IND 396/97	India	1997	Bovine	Sanyal et al.,2004	AF392941
289	IND 397/97	India	1997	Bovine	Sanyal et al.,2004	AF392942
290	IND 44/98	India	1998	Bovine	Sanyal et al.,2004	AF392943
291	IND 125/98	India	1998	Bovine	Sanyal et al.,2004	AF392928
292	IND 69/99	India	1999	Bovine	Sanyal et al.,2004	AF392947
293	IND 127/99	India	1999	Bovine	Sanyal et al.,2004	AF392929

294	IND 278/99	India	1999	Bovine	Sanyal et al.,2004	AF392934
295	IND 46/93	India	1993	Bovine	Sanyal et al.,2004	GQ220851
296	IND 36/95	India	1995	Bovine	Sanyal et al.,2004	GQ220852
297	IND 192/99	India	1999	Bovine	Sanyal et al.,2004	GQ220853
298	IND 235/99	India	1999	Buffalo	Sanyal et al.,2004	GQ220854
299	IND 367/00	India	2000	Bovine	Sanyal et al.,2004	GQ220855
300	IND 374/00	India	2000	Bovine	Sanyal et al.,2004	GQ220856
301	IND 377/00	India	2000	Bovine	Sanyal et al.,2004	GQ220857
302	IND 378/00	India	2000	Bovine	Sanyal et al.,2004	GQ220858
303	IND 379/00	India	2000	Bovine	Sanyal et al.,2004	GQ220859
304	IND 383/00	India	2000	Bovine	Sanyal et al.,2004	GQ220860
305	IND 384/00	India	2000	Bovine	Sanyal et al.,2004	GQ220861
306	IND 387/00	India	2000	Bovine	Sanyal et al.,2004	GQ220862
307	IND 51/01	India	2001	Bovine	Sanyal et al.,2004	GQ220863
308	IND 52/01	India	2001	Bovine	Sanyal et al.,2004	GQ220864
309	IND 107/01	India	2001	Bovine	Sanyal et al.,2004	GQ220865
310	IND 140/01	India	2001	Bovine	Sanyal et al.,2004	GQ220866
311	IND 148/01	India	2001	Bovine	Sanyal et al.,2004	GQ220867
312	IND 149/01	India	2001	Bovine	Sanyal et al.,2004	GQ220868
313	IND 152/01	India	2001	Bovine	Sanyal et al.,2004	GQ220869
314	IND 160/01	India	2001	Bovine	Sanyal et al.,2004	GQ220870
315	IND 248/01	India	2001	Bovine	Sanyal et al.,2004	GQ220871
316	IND 335/01	India	2001	Bovine	Sanyal et al.,2004	GQ220872
317	IND 342/01	India	2001	Bovine	Sanyal et al.,2004	GQ220873

318	IND 354/01	India	2001	Bovine	Sanyal et al.,2004	GQ220874
319	IND 373/01	India	2001	Bovine	Sanyal et al.,2004	GQ220875
320	IND 387/01	India	2001	Bovine	Sanyal et al.,2004	GQ220876
321	IND 388/01	India	2001	Bovine	Sanyal et al.,2004	GQ220877
322	IND 389/01	India	2001	Bovine	Sanyal et al.,2004	GQ220878
323	IND 396/01	India	2001	Bovine	Sanyal et al.,2004	GQ220879
324	IND 397/01	India	2001	Bovine	Sanyal et al.,2004	GQ220880
325	IND 423/01	India	2001	Bovine	Sanyal et al.,2004	GQ220881
326	IND 438/01	India	2001	Bovine	Sanyal et al.,2004	GQ220882
327	IND 61/02	India	2002	Bovine	Sanyal et al.,2004	GQ220883
328	Kimron/ISR/57	Israel	1957	NK	Carrillo et al., 2005	AY593797
329	ISR/3/63	Israel	1963	NK	Carrillo et al., 2005	AY593796
330	LEB/84	Lebanon	1984	Bovine	Carrillo et al., 2005	AY593799
331	PAK/1/54	Pakistan	1954	Buffalo	Carrillo et al., 2005	AY593795
332	YNBS/58	China	1958	NK	Chang et al., unpub. Data	AY390432
333	ZB/CHA/58	China	1958	NK	Xin et al., 2009	DQ533483
334	asia1leb83	Lebanon	1983	NK	Carrillo et al., 2005	AY593800
335	asia1leb-89	Lebanon	1989	NK	Carrillo et al., 2005	AY593798
336	IND_321/01	India	2002	cattle	Sanyal et al.,2004	AY687333
337	asia1-2isrl3-63	Israel	1963	NK	Carrillo et al., 2005	AY593796
338	LAO/1/96	Laos	1996	NK	Khounsy et al., 2009	EU667460
339	LAO/3/98	Laos	1998	Bovine	Khounsy et al., 2009	EU667461
340	IND 81/86	India	1986	Bovine	Mohapatra et al., 2008	DQ989306
341	IND 52/87	India	1986	Bubaline	Mohapatra et al., 2008	DQ989313

342	IND 21/89	India	1989	Bubaline	Mohapatra et al., 2008	DQ989316
343	IND 116/90	India	1990	Bovine	Mohapatra et al., 2008	DQ989305
344	IND 13/91	India	1991	Ovine	Mohapatra et al., 2008	DQ989312
345	IND 247/92	India	1992	Bovine	Mohapatra et al., 2008	DQ989307
346	IND 47/93	India	1993	Bovine	Mohapatra et al., 2008	DQ989315
347	IND 151/94	India	1994	Bovine	Mohapatra et al., 2008	DQ989303
348	IND 82/96	India	1996	Bovine	Mohapatra et al., 2008	DQ989309
349	IND_397-97	India	1997	Bovine	Mohapatra et al., 2008	DQ989308
350	IND 101/99	India	1999	Bovine	Mohapatra et al., 2008	DQ989310
351	IND 334/00	India	2000	Bovine	Mohapatra et al., 2008	DQ989304
352	IND 148/01	India	2001	Bovine	Mohapatra et al., 2008	DQ989317
353	IND 354/01	India	2001	Bovine	Mohapatra et al., 2008	DQ989314
354	IND 423/01	India	2001	Bovine	Mohapatra et al., 2008	DQ989319
355	IND 438/01	India	2001	Bovine	Mohapatra et al., 2008	DQ989321
356	IND 37/02	India	2002	Bubaline	Mohapatra et al., 2008	DQ989311
357	IND 61/02	India	2002	Bovine	Mohapatra et al., 2008	DQ989318
358	IND 139/02	India	2002	Bovine	Mohapatra et al., 2008	DQ989322
359	IND 182/02	India	2002	Bovine	Mohapatra et al., 2008	DQ989320
360	IND68/2012	India	2012	cattle	Subramaniam et al,.2013	KF570667
361	As/SIN/PAK/L2812/2009	Pakistan	2009	Buffalo	Jamal et al,. 2011	JN006721
362	As/SIN/PAK/L2810/2009	Pakistan	2009	Buffalo	Jamal et al,. 2011	JN006720
363	HKN_1/2005	Hong Kong	2005	NK	Xu et al., unpub. Data	HQ268512
364	As/KAB/AFG/L64	Afghanistan	2009	cattle	Jamal et al,. 2011	HQ439202

	2/009					
365	As/SIN/PAK/L5/2008	Pakistan	2008	Buffalo	Jamal et al,. 2011	JN006719
366	MAY/9/99	Malaysia	1999	cattle	Abdul-Hamid et al.,2011	HQ632774
367	Asia1/YS/CHA/05	China	2005	Bovine	Wang et al., 2011	GU931682
368	Nepal 29/97	Nepal	1997	NK	Brocchi et al., unpub. Data	EF134952
369	IND_97-03	India	2003	cattle	Mohapatra et al., 2008	DQ989323
370	TUR/1094/07/00	Turkey	2000	NK	Klein et al., 2006	DQ296529
371	IND/71/96	India	1996	cattle	Gurumurthy et al., 2002	AF390699
372	IND/316/94	India	1994	cattle	Gurumurthy et al., 2002	AF390688
373	IND/155/88	India	1988	cattle	Gurumurthy et al., 2002	AF390679
374	IND_141/02	India	2002	cattle	Sanyal et al.,2004	GQ220884
375	Asia1/IND/324/98	India	1998	Buffalo	Sanyal et al.,2004	AF392938
376	Asia1/IND/314/94	India	1994	cattle	Sanyal et al.,2004	AF392937
377	Asia1/IND/120/91	India	1991	cattle	Sanyal et al.,2004	AF392927
